# STRENGTHENING RETIREMENT PLANNING: A LONGITUDINAL EXAMINATION OF CONNECTEDNESS TO THE AGED FUTURE-SELF

**DOI:** 10.1093/geroni/igae098.2090

**Published:** 2024-12-31

**Authors:** Dannii Y Yeung, Gloria Lin, Alvin Ho

**Affiliations:** City University of Hong Kong, Hong Kong, China (People’s Republic); City University of Hong Kong, Hong Kong, China (People’s Republic); City University of Hong Kong, Hong Kong, China (People’s Republic)

## Abstract

The overall level of pre-retirement planning among working adults remains low. This study aims to identify effective strategies to strengthen younger and older workers’ preparation for retirement. A total of 401 Hong Kong Chinese workers (170 younger and 231 middle-aged; M=42.34, SD=11.15) were randomly allocated to one of four experimental conditions: future-self with a writing task (FSW), future-self only (FS), current-self with a writing task (CSW), and current-self only (CS). In the experiment, participants in FSW and FS conditions were presented with an aged-morphed photo of their future-self, while those in CSW and CS conditions were presented with a photo of their current-self. The experiment was followed by a 5-day diary study, in which the participants in FSW and CSW wrote down their plans to improve well-being of their future-selves in old age or in three months, respectively. Retirement planning in financial, health and psychosocial domains were assessed at two weeks (T1) and at six months (T2) after the experiment. Preliminary analyses show that participants in FS and FSW reported more negative emotions after viewing the future-self photo (F(3,396)=10.42, p<.001). A significant age group by condition interaction effect (F(3,317)=2.82, p=.039) was found in T1 retirement planning activities, with older workers in FSW reported more preparatory activities than younger workers in FSW, while such age effect was not observed in FS, suggesting that the 5-day writing task could strengthen one’s preparation for retirement. However, such age discrepancy effect diminished in T2. Implications on promotion of retirement planning will be discussed.

